# Attachment style dimensions are associated with neural activation during projection of mental states

**DOI:** 10.3389/fnhum.2022.899418

**Published:** 2022-08-04

**Authors:** Carlo Lai, Chiara Ciacchella, Daniela Altavilla, Giorgio Veneziani, Paola Aceto, Marco Cecchini, Massimiliano Luciani

**Affiliations:** ^1^Department of Dynamic and Clinical Psychology, and Health Studies, Sapienza University of Rome, Rome, Italy; ^2^Department of Philosophy, Communication and Performing Arts, Roma Tre University, Rome, Italy; ^3^Department of Emergency, Anesthesiological and Reanimation Sciences, Fondazione Policlinico Universitario A. Gemelli IRCCS, Rome, Italy; ^4^Department of Basic Biotechnological Sciences, Intensive Care and Perioperative Clinics, Catholic University of Sacred Heart, Rome, Italy; ^5^Department of Psychiatry, Catholic University of Sacred Heart, Rome, Italy

**Keywords:** attachment, brain correlates, sLORETA, Rorschach inkblots, projection

## Abstract

The aim of the present study was to investigate the association between attachment dimensions and neural correlates in response to the Rorschach inkblots. Twenty-seven healthy volunteers were recruited for the electroencephalographic registration during a visual presentation of the Rorschach inkblots and polygonal shapes. The Attachment Style Questionnaire (ASQ) was administered to participants. Correlations between the ASQ scores and standardized low-resolution brain electromagnetic tomography (sLORETA) intensities were performed. The Rorschach inkblots elicited several projective responses greater than the polygonal shapes (distortions, human and total movements, and embellishments). Only during the Rorschach inkblots presentation, discomfort with closeness and relationships as secondary subscales were negatively correlated with the activation of right hippocampus, parahippocampus, amygdala, and insula; need for approval subscale was negatively correlated with the activation of orbital and prefrontal cortex and left hippocampus. Moreover, the correlations between attachment dimensions and neural activation during the Rorschach inkblots were significantly higher compared to the same correlations in response to polygonal shapes. These findings suggest that attachment style can modulate brain activation during the projective activity of the Rorschach inkblots.

## Introduction

Projection is a psychodynamic mechanism where the subject’s own internal states are attributed to the external environment. The projective tests are psychological tools with intentionally ambiguous visual stimuli, and the subject’s task is to provide a description inspired by the represented image. This task could bring out unconscious psychic contents, such as hidden emotions and internal conflicts. Previously, after a long debate on terms “objective” vs. “projective” as personality tests descriptors (Meyer and Kurz, 2006), it has been stated that other terms as “self-report inventories” vs. “performance tasks” should be considered as an alternative. However, in the present study, the use of the term “projection” necessarily refers to a specific psychodynamic process rather than a specific test descriptor (Meyer and Kurz, 2006).

The Rorschach Inkblot Method (RIM) (Rorschach, 1921) is the most used tool for the assessment of several aspects of the personality (Exner, 1989; Lerner, 1990; Berant et al., 2005; Weiner, 2018). There are many methods for administering and interpreting the RIM that have been developed in the one-century history. Nowadays, Exner’s Comprehensive System (Exner, 2003) is one of the most investigated methods. Mihura et al. (2013) stated that, although historically, the Rorschach inkblots have been classified as a “projective” test, and contemporary psychologists do not associate the test method with projection (Exner, 1989; Meyer and Kurz, 2006; Bornstein, 2007; McGrath, 2008). According to Exner (1989), this seems to be true in the first phase of attribution of meaning to the inkblots, where global responses are often common and most likely related to a perceptive rather than projective process. However, in the subsequent phases, the existing ambiguity among the stimuli allows some of the stronger subject needs, sets, and attitudes to become influential during stimuli translation (Exner, 1989). Moreover, Exner (1989) hypothesized that the presence of specific indicators in the RIM responses suggests a projective activity. These indicators show that an extrapolation occurred beyond the real or objective attributes of the inkblots (Pianowski et al., 2016) as perceptual distortions, attributions of occurring movements, and illogical or thematically relevant embellishments (Exner, 1989; Weiner, 2003). The Comprehensive System (Exner, 2003) stated that these three indicators are the best markers of projective material (Weiner, 2003).

Moreover, recent studies have highlighted the implementation of the coding system and interpretation of the RIM and have developed more sophisticated empirical guidelines for clinical assessment (Meyer et al., 2011; Mihura et al., 2013).

The attachment theory showed how the internalization of different aspects of a child’s relational experience may influence behavioral and emotional regulation in adulthood. In particular, early negative relational experiences could lead to emotional regulation problems in adolescence, which could determine the onset of various negative clinical outcomes from early adolescence, such as suicidality and affective disorders (Solano et al., 2016). Interestingly, abnormalities have been documented in the brains of adolescents with affective disorders (Serafini et al., 2014). In particular, a previous review reported reductions in the volume of basal ganglia and the hippocampus in adolescent with unipolar depression, whereas reduced corpus callosum volume and increased rates of deep white matter hyperintensities were found in adolescent bipolar depression (Serafini et al., 2014). The attachment styles could be conceptualized as a continuum of two dimensions, namely, avoidance and anxiety, rather than by categories, as previously proposed (Mikulincer and Shaver, 2007; Shaver and Mikulincer, 2008). Subjects with higher levels of anxiety are afraid of rejection, separation, and abandonment, whereas subjects with higher levels of avoidance feel discomfort with intimacy and difficulty in depending on others (Shaver and Mikulincer, 2008).

In literature, an association between attachment dimensions and personality characteristics detected by the RIM scores has been demonstrated; specifically, it has been found that the attachment dimensions were associated with texture response (Cassella and Viglione, 2009). Berant et al. (2005) demonstrated that the levels of anxious attachment were positively associated with the difficulties in regulating and controlling emotions and self-perceptions of being relatively helpless and unworthy measured by the Rorschach scores. Moreover, the levels of avoidant attachment were positively associated with lack of acknowledgment of need states and maintenance of a grandiose self, measured in the RIM. This result was interpreted as the dynamic manifestation of hyperactivation and deactivation strategies (Berant et al., 2005). Moreover, a previous study showed that, during the Rorschach inkblots, the dimensions of anxious attachment were positively associated with the use of projective identification, penetration, incongruous combination, and fabulized combination, whereas the dimensions of avoidant attachment were positively associated with the use of the devaluation (Berant and Wald, 2009).

In a recent study (Cecchini et al., 2015), attachment dimensions showed a significant association with brain activity during a social visual task. Interestingly, another study showed that the different styles of attachment can be associated with biases in selective attention toward emotional information of the environment (Dan and Raz, 2012).

Neurobiological studies showed that the limbic system is a complex set of cerebral areas, including the hippocampus, the amygdala, the insula, and several other nearby areas, and it appears to be primarily responsible for emotional processes and memory formation (Rolls, 2015). The subjects with avoidant attachment style showed a high activation of the prefrontal cortex, anterior cingulate, and amygdala in response to negative social images during both the spontaneous vision and during cognitive revaluation, while the subjects with anxious attachment showed a high activation of the amygdala and parahippocampus only during a spontaneous vision of the images (Mikulincer and Shaver, 2007; Vrtička et al., 2012). The authors interpreted these findings stating that subjects with avoidant attachment seem to use less efficient revaluation strategies for regulating negative emotions, i.e., they prefer to use avoidance and emotional suppression strategies (Mikulincer and Shaver, 2007; Vrtička et al., 2012).

Asari et al. (2010a,b) have demonstrated the involvement of the amygdala in the modulation of frontotemporal connectivity suggesting the interference of emotional effects during the Rorschach inkblots. Moreover, previous studies showed that human movement responses to the Rorschach inkblots were associated with mirroring activity (Giromini et al., 2010; Pineda et al., 2011; Porcelli et al., 2013). A recent neurobiological study (Luciani et al., 2014) showed that the presentation of the Rorschach inkblots, compared to polygonal shapes, involved later frontal and parietal activities, during the meaning attribution of the stimuli. This finding could reflect the projective activity in response to the Rorschach inkblots. These results seemed to confirm Exner’s study (1989) about the probability that the projective process occurs after the primary common global response.

The aim of the present study was to investigate the association between attachment dimensions and neural correlate in response to the Rorschach inkblots and to continue, through a sample expansion, a previous study of Luciani et al. (2014) where late higher brain activity was shown in response to the Rorschach inkblots compared to the polygonal shapes. In the present study, hypotheses were that dimensions of avoidance attachment will be negatively associated with activation of the limbic areas and that dimensions of anxiety attachment will be positively associated with activation of the frontolimbic circuits, during projective activity in response to the Rorschach inkblots compared to the polygonal shapes.

## Materials and methods

### Participants

The participants answered a public announcement that required free and voluntary participation in the study. The inclusion criteria were: age between 20 and 50 years, correct vision, and high school graduation. Subjects with diagnosed neurological and psychiatric disorders, drug use, and those who affirmed to have performed or studied the RIM were excluded. Thirty healthy volunteers were recruited. After cleaning electroencephalographic (EEG) data (see below), 27 subjects (5 males and 22 females, mean age = 24.4 ± 5.6 years) were included in the statistical analysis. All the subjects were asked to sign the informed consent. The study was carried out in Clinical Neuroscience Laboratory of the Department of Dynamic and Clinical Psychology, and Health Studies, Sapienza University of Rome. The ethical committee of the same Department approved the present research project.

### Procedure

The visual stimuli consisted of the 10 gray Rorschach inkblots and 10 gray polygonal shapes on white backgrounds. The choice to use stimuli with different degrees of graphical structure (Rorschach inkblots vs. polygonal shapes) was done to differentiate and to compare the different degrees of projective activity (with many meanings perceived in the Rorschach inkblots vs. few meanings perceived in the polygonal shapes) among the participants, as observed in a previous study (Luciani et al., 2014). The choice to use a gray version was done to match the two types of stimuli for colors and shades (the Rorschach inkblots and polygonal shapes).

The images were created in Corel Photo-paint 12 without shadows and contour lines to match for contrast, luminance, and brightness. The stimuli measured roughly 520 × 400 pixels on the screen.

Before the EEG registration, for the attachment assessment, the self-report Attachment Style Questionnaire (ASQ) (Feeney et al., 1994) was administered to participants. The ASQ (40 items) was designed to measure five dimensions of adult attachment: “Confidence” (8 items), “Discomfort with closeness” (10 items), “Relationships as secondary” (7 items), “Need for approval” (7 items), and “Preoccupation with relationships” (8 items). Each item is rated on a 6-point scale. The psychometric values of reliability and validity of the ASQ in our sample of 27 subjects are given in Table 1. The values of reliability and validity found in the present study confirmed the values reported in the literature (Clark and Watson, 1995; Fossati et al., 2003, 2007).

**TABLE 1:** Mean, SD, Cronbach’s alpha, average interitem correlation, and split half ρ related to the five dimensions of the Attachment Style Questionnaire.

	*M*	*SD*	Cronbach α	Average inter-item correlation	Split half ρ*
Confidence	33.60	3.9	0.65	0.20	0.52
Discomfort with closeness	33.52	6.2	0.70	0.21	0.83
Relationships as secondary	13.48	4.9	0.85	0.49	0.86
Need for approval	16.93	4.7	0.80	0.38	0.87
Preoccupation with relationships	26.96	5.2	0.70	0.25	0.69

*Spearman-Brown correction.

**FIGURE 1:**
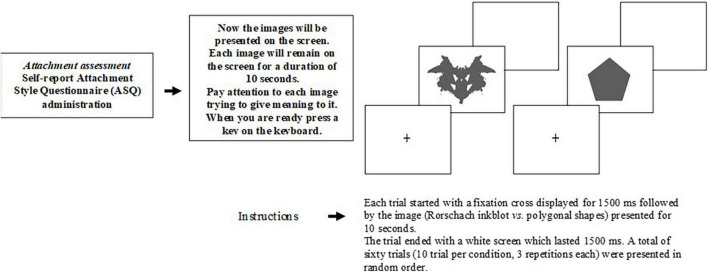
The procedure of experimental visual task.

Participants were seated at a viewing distance of 80 cm from a PC monitor (27 cm, 75–Hz, 1,024 × 768). The stimuli were presented using E-Prime (version 2.0.8.90; Psychology Software Tools Incorporation; Pittsburgh, Philadelphia, United States). Each trial started with a fixation cross displayed for 1,500 ms, followed by the stimulus (Rorschach inkblots vs. polygonal shapes) presented for 10 s. The trial ended with a white screen, which lasted for 1,500 ms. A total of sixty trials (10 trials *per* condition, 3 repetitions each) were presented in a random order (Figure 1). The instruction for the participants was to pay attention to the stimuli and to think about the possible meaning of each image. The duration of the EEG visual task was about 20 min (Luciani et al., 2014). At the end of the procedure, every participant was asked to report the content of responses that he/she had identified for each presented stimulus (sixty presentations). In order to evaluate projective material (Weiner, 2003), the responses to each presented stimulus were coded by a trained psychologist for the following indicators: distortion (minus formal quality); movement (human, animal, and inanimate); and embellishment (deviant verbalization, deviant response, peculiar logic, incongruous combination, fabulized combination, contamination, morbid content, cooperative movement, and aggressive movement).

### Electroencephalogram recording and analysis

Data were continuously recorded at 250 Hz using NetStation 4.5.1 with 256 channels HydroCel Geodesic Sensor Net referenced to the vertex (Cz). Impedances were kept below 40 kΩ. The data were digitally filtered (30 Hz low pass) offline. Net Station artifacts detection settings were set at 200 μV for bad channels, 150 μV for eye blinks, and 100 μV for eye movements. Segments containing eye blinks, eye movements, or more than 15 bad channels were excluded. After applying EEG editing procedures, the data of three participants were excluded due to the presence of artifacts. The segmentation epoch duration was from 100 ms before to 1,500 ms after stimulus onset with baseline correction at -100 ms of stimulus onset. For data analysis, six-time windows were selected: from 50 to 123 ms for the P100, from 123 to 203 ms for N170, three intervals (LC1-LC2-LC3) of 100 ms, and one interval (LC4) of 500 ms from 500 to 1,000 ms.

### Source analyses (standardized low-resolution brain electromagnetic tomography)

Brain sources, describing the neural sources of the measured scalp potentials, were estimated with GeoSource software (version 2.0; EGI, Eugene, Oregon, United States), which performed the RMI data normalization and extraction for each subject. Source locations were derived from the probabilistic map of the MNI305 average (Montreal Neurological Institute 305 subjects). Based on the probabilistic map, gray matter volume was parcellated into 7-mm voxels; each voxel served as a source location with three orthogonal orientation vectors. This resulted in a total of 2,447 source triplets whose anatomical labels were estimated using the Talairach Daemon (Cecchini et al., 2013; Lai et al., 2020; Altavilla et al., 2021). For both the conditions (Rorschach inkblots vs. polygonal shapes), mean intensity (nA) was obtained for each time window (P100, N170, LC1, LC2, LC3, and LC4) on all the Brodmann areas (BAs) by doing the square root of the sum of the three dipoles using the minimum norm least squares method (Electrical Geodesics Inc., 2007). For each time window, nA was calculated on frontal (BA: 4-6-8-9-10-11-44-45-46-47), parietal (BA: 1-2-3-5-7-39-40-43), temporal (BA:20-21-22-37-38), cingulate (BA: 23-24-25-29-30-31-32-33), and limbic areas (amygdala, hippocampus, BA: 13-28-34-35-36).

### Statistical analyses

The paired *t*-test between the responses to the Rorschach inkblot and polygonal shapes on distortion (minus formal quality); movement (human, animal, and inanimate); and embellishment (deviant verbalization, deviant response, peculiar logic, incongruous combination, fabulized combination, contamination, morbid content, cooperative movement, and aggressive movement) has been performed in order to evaluate the projective processes (Weiner, 2003). Data transformation (*2* × *Arcosin*√*x*) was performed when appropriate.

Paired *t*-tests with Bonferroni correction were performed between mean intensities during the Rorschach inkblots vs. polygonal shapes presentations in each time window for each BA of frontal (BA: 4-6-8-9-10-11-44-45-46-47), parietal (BA: 1-2-3-5-7-39-40-43), temporal (BA: 20-21-22-37-38), cingulate (BA: 23-24-25-29-30-31-32-33), and limbic areas (amygdala, hippocampus, and parahippocampus BA: 13-28-34-35-36).

Correlation analyses (Pearson correlation) were performed between scores of each ASQ dimension and mean intensities of each BA during each condition for every interval (Bonferroni correction was applied) in order to test the association between attachment scores and brain intensities in response to structured and non-structured conditions. Each significant correlation obtained between scores of the ASQ dimensions and BA intensities in response to one of the two conditions (Rorschach inkblots and polygonal shapes) was statistically compared with the same correlation obtained in response to the other one by using the procedure described by Meng et al. (1992). The *p*-value accepted was *p* < 0.05.

All the statistical analyses were performed with Statistica 8 (Weiß, 2007).

## Results

The Rorschach inkblots elicited a number of total responses greater than the polygonal shapes [18.4 ± 4.1 vs. 13.7 ± 3.8; *t*(26) = 5.3; *p* < 0.001]. Moreover, the Rorschach inkblots elicited a number of projective responses greater than the polygonal shapes [distortion: 0.9 ± 1.1 vs. 0.1 ± 0.4; *t*(26) = 3.1; *p* < 0.001; human movement: 3.5 ± 1.9 vs. 0.0 ± 0.0; *t*(26) = 9.5; *p* < 0.001; total movement: 4.2 ± 2.0 vs. 0.0 ± 0.0; *t*(26) = 10.8; *p* < 0.001; embellishment: 0.9 ± 1.1 vs. 0.2 ± 0.6; *t*(26) = 2.8; *p* = 0.010; and total projective responses: 6.0 ± 2.9 vs. 0.4 ± 0.9; *t*(26) = 8.9; *p* < 0.001]. The percentage of projective responses on the total responses was greater on the Rorschach inkblots than on the polygonal shapes [34 vs. 3%; *t*(26) = 10.3; *p* < 0.001].

The mean scores of each attachment dimension for the 27 participants were the following: confidence (mean: 33.6 ± 3.9), discomfort with closeness (mean: 33.6 ± 6.2), relationships as secondary (mean: 13.5 ± 5.0), need for approval (mean: 16.9 ± 4.7), and preoccupation with relationships (mean: 27.0 ± 5.2).

As shown in Table 2, paired *t*-test showed a lower activation during the Rorschach inkblots compared to polygonal shapes of left amygdala, hippocampus, BA28, BA34, BA35, BA36 at P100, and N170, and left BA38 at P100 (0.001 < *p* < 0.042). Differently, a greater activation was found during the Rorschach inkblots compared to polygonal shapes of left and right BA11, BA25, BA28, right amygdala, BA34, BA38, from LC1 to LC3 (0.001 < *p* < 0.002); of left amygdala, BA34, BA38, right hippocampus, BA20, BA35, BA36, and BA47 at LC2 (0.001 < *p* < 0.005); of left BA34 at LC3 (*p* < 0.001); and, finally, of left and right BA11 at LC4 (*p* = 0.049; *p* < 0.001).

**TABLE 2:** Paired *t*-test between the mean intensity during the Rorschach inkblots vs. polygonal shapes for each left (l) and right (r) frontal (BA: 4-6-8-9-10-11-44-45-46-47), parietal (BA: 1-2-3-5-7-39-40-43), temporal (BA: 20-21-22-37-38), cingulate (BA: 23-24-25-29-30-31-32-33), and limbic areas (amygdala, hippocampus, BA: 13-28-34-35-36) in each interval: P100, N170, LC1, LC2, LC3, and LC4 (Bonferroni correction was applied).

Intervals	Brodmann area (BA)	
P100	lAmygdala*^p^*^= 0.012^; lHippocampus*^p^*^= 0.020;^ lBA28*^p^*^= <0.001^; lBA34*^p^*^= 0.042^; lBA35*^p^*^= <0.001^; lBA36*^p^*^= <0.001^ lBA38*^p^*^= <0.001^	Polygonal shapes > Rorschach inkblots
N170	lAmygdala*^p^*^= <0.001^; lHippocampus*^p^*^= 0.005^; lBA28*^p^*^= <0.001^; lBA34*^p^*^= 0.028^; lBA35*^p^*^= <0.001^ lBA36*^p^*^= <0.001^	Polygonal shapes > Rorschach inkblots
LC1	rAmygdala*^p^*^= <0.001^; l&rBA11*^p^*^= <0.001 &^*^p^*^= <0.001^; l&rBA25*^p^*^= <0.001 &^*^p^*^= <0.001^; l&rBA28*^p^*^= <0.001&^*^p^*^= <0.001^ rBA34*^p^*^= <0.001^; rBA38*^p^*^= <0.001^	Rorschach inkblots > Polygonal shapes
LC2	l&rAmygdala*^p^*^= <0.001 &^*^p^*^= <0.001^; rHippocampus*^p^*^= <0.001^; l&rBA11*^p^*^= <0.001 &^*^p^*^= <0.001^; rBA20*^p^*^= <0.001^ l&rBA25*^p^*^= <0.001 &^*^p^*^= <0.001^; l&rBA28*^p^*^= <0.001 &^ *^p^*^= <0.001^; l&rBA34*^p^*^= <0.001&^*^p^*^= <0.001^; rBA35*^p^*^= <0.001^ rBA36*^p^*^= <0.001^; l&rBA38*^p^*^= 0.005 &^*^p^*^= <0.001^; rBA47*^p^*^= <0.001^	Rorschach inkblots > Polygonal shapes
LC3	rAmygdala*^p^*^= <0.001^; l&rBA11*^p^*^= <0.001 &^*^p^*^= <0.001^; l&rBA25*^p^*^= <0.001 &^*^p^*^= <0.001^; l&rBA28*^p^*^= 0.002&^*^p^*^= <0.001^ l&rBA34*^p^*^= <0.001 &^*^p^*^= <0.001^; rBA38*^p^*^= <0.001^	Rorschach inkblots > Polygonal shapes
LC4	l&rBA11*^p^*^= 0.049 &^*^p^*^= <0.001^	Rorschach inkblots > Polygonal shapes

As shown in Table 3, during the presentation of the Rorschach inkblots, discomfort with closeness was negatively correlated with the activation of right hippocampus (*r* = –0.51; *p* = 0.003; *d* = 1.19) and of right parahippocampus (BA35, *r* = –0.50; *p* = 0.004; *d* = 1.15) at LC3; relationships as secondary was negatively correlated with activation of right parahippocampus at LC1 (BA34, *r* = –0.54; *p* = 0.002; *d* = 1.28), LC2 (BA28, *r* = –0.50; *p* = 0.004; *d* = 1.15; BA34 *r* = –0.56; *p* = 0.001; *d* = 1.35), LC3 (BA34, *r* = –0.56; *p* = 0.001; *d* = 1.35), and LC4 (BA34, *r* = –0.54; *p* = 0.001; *d* = 1.28); with activation of right amygdala at LC2 (*r* = –0.49; *p* = 0.004; *d* = 1.12) and LC3 (*r* = –0.49; *p* = 0.004; *d* = 1.12); and with activation of right insula (BA13, *r* = –0.53; *p* = 0.002; *d* = 1.25) at LC3; need for approval was negatively correlated with the activation of right anterior prefrontal cortex (BA10) at LC2 (*r* = –0.60; *p* = 0.001; *d* = 1.50), LC3 (*r* = –0.66; *p* < 0.001; *d* = 1.76) and LC4 (*r* = –0.59; *p* = 0.001; *d* = 1.46); with activation of left and right orbitofrontal cortex (BA11, *r* = –0.54; *p* = 0.003; *d* = 1.28; *r* = –0.57; *p* = 0.002; *d* = 1.39) at LC4 and with activation of left hippocampus (*r* = –0.52; *p* = 0.006; *d* = 1.22) at LC3.

**TABLE 3:** Comparisons between the correlations (Pearson correlation), the ASQ dimensions (confidence, discomfort with closeness, relationships as secondary, need for approval, and preoccupation) scores and the mean activation of Brodmann areas (BAs) in response to the Rorschach inkblot vs. polygonal shapes.

Intervals		Correlations between attachment dimensions and brain activity in response to		Correlations between brain activity in response to Rorschach inkblot and to polygonal shapes	*Z*-values
		Rorschach inkblot	Polygonal shapes		
**ASQ discomfort with closeness**					
LC3 (400–500 ms)	rHippocampus rBA35	*r* = –0.51; *p* = 0.003 *r* = –0.50; *p* = 0.004	*r* = –0.13; *p* = 0.511 *r* = –0.12; *p* = 0.539	*r* = 0.66; *p* < 0.001 *r* = 0.69; *p* < 0.001	*z* = –2.42; *p* = 0.008 *z* = –2.51; *p* = 0.006
**ASQ relationships as secondary**					
LC1 (200–300 ms)	rBA34	*r* = –0.54; *p* = 0.002	*r* = –0.08; *p* = 0.692	*r* = 0.55; *p* = 0.003	*z* = –2.55; *p* = 0.005
LC2 (300–400 ms)	rAmygdala rBA28 rBA34	*r* = –0.49; *p* = 0.004 *r* = –0.50; *p* = 0.004 *r* = –0.56; *p* = 0.001	*r* = –0.23; *p* = 0.248 *r* = –0.23; *p* = 0.254 *r* = –0.29; *p* = 0.141	*r* = 0.69; *p* < 0.001 *r* = 0.69; *p* < 0.001 *r* = 0.61; *p* = 0.001	*z* = –1.76; *p* = 0.039 *z* = –1.83; *p* = 0.034 *z* = –1.70; *p* = 0.045
LC3 (400–500 ms)	rAmygdala rBA13 rBA34	*r* = –0.49; *p* = 0.004 *r* = –0.53; *p* = 0.002 *r* = –0.56; *p* = 0.001	*r* = –0.26; *p* = 0.186 *r* = –0.22; *p* = 0.263 *r* = –0.34; *p* = 0.084	*r* = 0.72; *p* < 0.001 *r* = 0.54; *p* = 0.003 *r* = 0.69; *p* < 0.001	*z* = –1.65; *p* = 0.049 *z* = –1.75; *p* = 0.040 *z* = –1.57; *p* = 0.058
LC4 (500–1,000 ms)	rBA34	*r* = –0.54; *p* = 0.001	*r* = –0.24; *p* = 0.222	*r* = 0.59; *p* = 0.001	*z* = –1.81; *p* = 0.036
**ASQ need for approval**					
LC2 (300–400 ms)	rBA10	*r* = –0.60; *p* = 0.001	*r* = –0.23; *p* = 0.253	*r* = 0.66; *p* < 0.001	*z* = –2.48; *p* = 0.007
LC3 (400–500 ms)	rBA10 lHippocampus	*r* = –0.66; *p* < 0.001 *r* = –0.52; *p* = 0.006	*r* = –0.18; *p* = 0.364 *r* = –0.32; *p* = 0.105	*r* = 0.46; *p* = 0.020 *r* = 0.81; *p* < 0.001	*z* = –2.63; *p* = 0.004 *z* = –1.77; *p* = 0.038
LC4 (500–1,000 ms)	rBA10 lBA11 rBA11	*r* = –0.59; *p* = 0.001 *r* = –0.54; *p* = 0.003 *r* = –0.57; *p* = 0.002	*r* = –0.10; *p* = 0.607 *r* = –0.10; *p* = 0.628 *r* = –0.04; *p* = 0.840	*r* = 0.44; *p* = 0.020 *r* = 0.47; *p* = 0.010 *r* = 0.49; *p* = 0.009	*z* = –2.50; *p* = 0.006 *z* = –2.26; *p* = 0.012 *z* = –2.77; *p* = 0.003

Confidence and preoccupation with relationships did not show any significant correlations during the presentation of the Rorschach inkblots (see Table 3).

**FIGURE 2:**
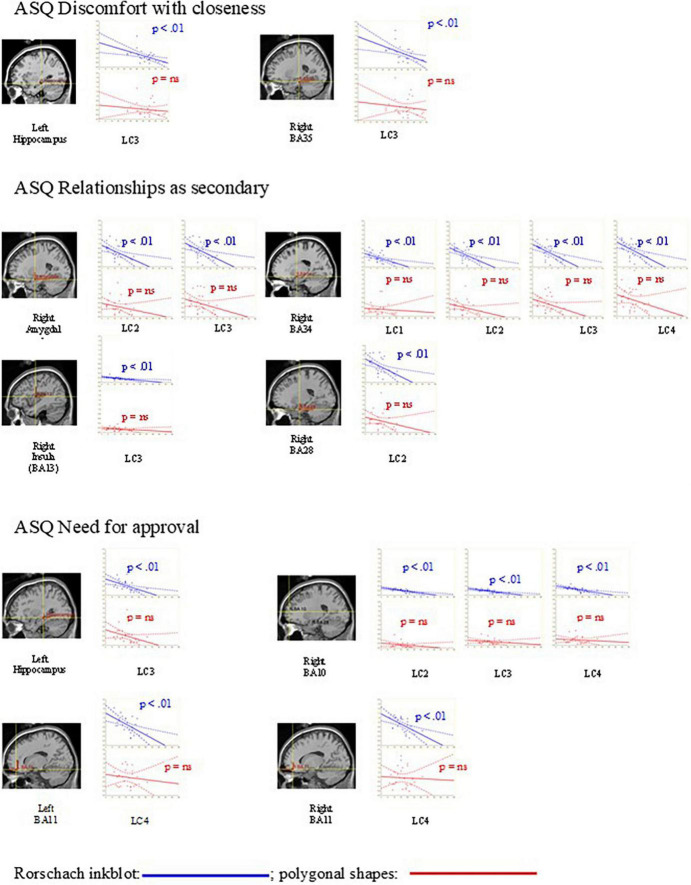
Scatterplot graphs between attachment dimensions (ASQ discomfort with closeness; ASQ relationships as secondary; and ASQ need for approval) and brain activations in response to the Rorschach inkblots and polygonal shapes. As it is also reported in Table 2, many associations between attachment dimensions and brain response to the Rorschach inkblots from 200 ms (LC1: 200–300; LC2: 300–400; LC3: 400–500; and LC4: 500–1,000) were significant (*p* < 0.01), while there were no significant associations between attachment dimensions and brain response to polygonal shapes (*p* = ns).

No significant correlation between the attachment dimensions and brain intensities was found during the presentation of the polygonal shapes. Moreover, as shown in Table 3, the number of correlations between attachment dimensions and neural activation during the Rorschach inkblots (mean *r*: –0.54 ± 0.05) was significantly higher (mean *z* = 2.06 ± 0.42; monodirectional critical *z* = 1.64; *p* = 0.05) compared to the same correlations in response to polygonal shapes (mean *r*: –0.19 ± 0.09). In Figure 2, the scatterplot graphs between attachment dimensions and brain activations during the presentation of the Rorschach inkblots and polygonal shapes are shown. As given in Table 3, several correlations between attachment dimensions and brain in response to the Rorschach inkblot were significant (*p* < 0.01), whereas there were no significant associations between attachment dimensions and brain response to polygonal shapes (*p* > 0.08).

## Discussion

The main findings of the present study were that avoidance attachment dimensions (specifically the discomfort with closeness and the relationships as secondary) were negatively associated with the activation of the right hippocampus, parahippocampus (BA28, BA34, and BA35), amygdala, and insula (BA13) and that dimension of anxious attachment (need of approval) was negatively associated with right prefrontal (BA10), left and right orbitofrontal (BA11), and left hippocampus, during projective activity in response to the Rorschach inkblots and not to polygonal shapes (see Figure 2). The greater number of projective responses during the Rorschach inkblots compared to the polygonal shapes suggests that the Rorschach inkblots elicited a greater projective activity, as observed in a previous study (Luciani et al., 2014).

Moreover, a greater activation during polygonal shapes compared to the Rorschach inkblots of left limbic (amygdala, hippocampus, BA28, BA34, BA35, and BA36), and temporal (BA38) areas were found before 200 ms, whereas a greater activation of right limbic (amygdala, hippocampus, and BA34) and temporal (BA38) areas and of bilateral frontal (BA11) and cingulate (BA25) areas during the Rorschach inkblots compared to polygonal shapes was found after 200 ms. This finding confirmed results from a previous study showing that the participants performed two different mental processes with the Rorschach inkblots and polygonal shapes (Luciani et al., 2014).

Even though there was a strong association between attachment dimensions and brain activity in response to both the stimuli, the association found in response to the Rorschach inkblots was significantly higher compared to the association found in response to the polygonal shapes. This finding showed that attachment dimensions modulated projective response only in response to a non-structured stimulus such as the Rorschach inkblots. A possible explanation of this result could be that less structured stimulus such as the Rorschach inkblots facilitated the projection of deep characteristics of the personality as attachment styles. Coherently with the findings of Exner (1989), where the projective process seemed to occur late in the subsequent phases of the common global response, and with Luciani et al. (2014) study where a greater and late projective activity was revealed in response to the Rorschach inkblots, in the present study, the attachment dimensions were correlated with late brain activity (>200 ms.). These results suggested that the late projective response to unstructured visual stimuli could be the expression of the deep characteristics of the participant’s personality.

In literature, it has been demonstrated the involvement of the amygdala, parahippocampus, and insula in implicit memory (Suzuki, 1996), stressful social situations (Foley and Kirschbaum, 2010), and emotional processing (Koban et al., 2010; Lamm et al., 2011). Accordingly to other studies, deactivation of these areas suggested the use of emotional suppression in subjects with high scores of avoidance (Kim and Hamann, 2007; Mikulincer and Shaver, 2007; McRae et al., 2010; Vrtička et al., 2011; Vrtička and Vuilleumier, 2012).

In the present study, the items related to the two dimensions of avoidance attachment showed clear differences, while discomfort with closeness suggested only the presence of discomfort inside the relationships, relationships as secondary showed an apparent devaluation of the relationships. Specifically, the relationships as secondary was negatively associated with a more wide and stable activation of the right limbic areas in response to the Rorschach inkblots compared to discomfort with closeness. This result suggested that the attitude to a devaluation of the relationships is the dimension of the avoidance attachment that may have a pivotal role during the projective activity. This finding is coherent with a previous study where the avoidance attachment score was positively associated only with the use of the devaluation assessed throughout the Rorschach inkblots (Berant and Wald, 2009).

The need for approval was the only anxious attachment dimension that showed an association with the neural activity in response to the Rorschach inkblots. However, in contrast with the hypothesis, this dimension was negatively correlated with the activation of the left and right orbital and prefrontal cortex (BA10, BA11) and left hippocampus. Previous studies showed a hyperactivation of the frontolimbic circuits in the subjects with higher levels of anxious attachment (Vrtička and Vuilleumier, 2012). A possible explanation of the findings dissonance of the present study could be found in the task proposed to the participants that lack explicit emotional valence stimuli. However, the negative association between the need for approval and orbital and prefrontal cortex sustained the idea that anxious attachment is negatively associated with the levels of mentalization (Fonagy and Luyten, 2009; Hünefeldt et al., 2013). Mentalization is a process involved in the representation of internally focused information about the environment and it is positively associated with the frontal cortex activity (Fonagy and Luyten, 2009). Moreover, the left orbitofrontal cortex and left hippocampus are specifically linked to autobiographic memories (Gilboa, 2004; Lieberman, 2007). The fact that higher levels of anxious attachment were associated with the lower orbital and prefrontal cortex activity sustains the hypothesis about an inhibition of the involvement of internal representations and recovery of past experiences in anxiously attached participants during projective activity, coherently with recent findings (Fonagy and Luyten, 2009; Hünefeldt et al., 2013).

The limitation of the present study was that in the experimental task, the 10 Rorschach inkblots were presented as gray and without colors and shades. It could be interesting to test whether the effect is confirmed or increased in front of the original Rorschach inkblots (with colors and shades). Indeed, it has been found that colors and the vividness of an image could influence the brain response to visual stimuli (Yoto et al., 2007). Moreover, considering the association between the EEG activity during the Rorschach inkblots and the attachment dimensions scores, it could be interesting to evaluate the association between brain activity during the Rorschach inkblots and other unrelated domains or constructs (divergent validity). Moreover, it could be interesting to consider a third set of stimuli formed by similar high perceptual and cognitive complexity shapes as a control in order to evaluate a possible effect of stimuli complexity. Finally, the small number of participants and the presence of more women than men should be considered as limitations. In this regard, a recent study highlighted the presence of sex-related differences in brain activities of women and men with avoidant attachment style (Altavilla et al., 2021). Further studies should include a more balanced sample with a larger number of participants to allow generalization of results.

## Conclusion

In conclusion, this study showed a negative association between avoidance attachment and intensities of right limbic areas (parahippocampus, amygdala, and insula) and a negative association between anxious attachment and intensities of frontolimbic circuits (left and right orbital and prefrontal cortex and left hippocampus), during late projective activity in front of the Rorschach inkblots that lacks in front of the polygonal shapes. These findings showed as attachment style that could modulate brain activation of the projective activity in response to non-structured stimuli during the administration of the Rorschach inkblots. From a clinical point of view, it should be taken into account that the relationship devaluation mechanism that characterizes individuals with avoidant attachment might modulate the projective activities that occur during psychological assessment. Furthermore, in working with individuals with anxious attachment style, it should be considered that projective activity could be influenced by difficulties in mentalization and inhibition of the involvement of internal representations. Further studies could investigate this effect also on clinical sample.

## Data availability statement

The raw data supporting the conclusions of this article will be made available by the authors, upon reasonable request.

## Ethics Statement

The studies involving human participants were reviewed and approved by the Department of Dynamic and Clinical Psychology, and Health Studies, Sapienza University of Rome. The patients/participants provided their written informed consent to participate in this study.

## Author contributions

CL, DA, PA, MC, and ML contributed to the conception and design of the study. DA organized the database. CL and DA performed the statistical analysis. CL, CC, DA, and GV wrote the draft of the manuscript. All authors contributed to manuscript revision, read, and approved the submitted version.

## References

[B1] Altavilla D. Ciacchella C. Pellicano G. R. Cecchini M. Tambelli R. Kalsi N. (2021). Neural correlates of sex-related differences in attachment dimensions. *Cogn. Affect. Behav. Neurosci.* 21 191–211. 10.3758/s13415-020-00859-5 33560494 PMC7994245

[B2] Asari T. Konishi S. Jimura K. Chikazoe J. Nakamura N. Miyashita Y. (2010a). Amygdalar modulation of frontotemporal connectivity during the inkblot test. *Psychiatry Res.* 182 103–110. 10.1016/j.pscychresns.2010.01.002 20456928

[B3] Asari T. Konishi S. Jimura K. Chikazoe J. Nakamura N. Miyashita Y. (2010b). Amygdalar enlargement associated with unique perception. *Cortex* 46 94–99. 10.1016/j.cortex.2008.08.001 18922517

[B4] Berant E. Mikulincer M. Shaver P. R. Segal Y. (2005). Rorschach correlates of self-reported attachment dimensions: Dynamic manifestations of hyperactivating and deactivating strategies. *J. Pers. Assess.* 84 70–81. 10.1207/s15327752jpa8401_13 15639770

[B5] Berant E. Wald Y. (2009). Self-Reported attachment patterns and Rorschach-related scores of ego boundary, defensive processes, and thinking disorders. *J. Pers. Assess.* 91 365–372. 10.1080/00223890902936173 20017066

[B6] Bornstein R. F. (2007). Might the Rorschach be a projective test after all? Social projection of an undesired trait alters Rorschach Oral Dependency scores. *J. Pers. Assess.* 88 354–367. 10.1080/00223890701333514 17518556

[B7] Cassella M. J. Viglione D. J. (2009). The Rorschach texture response: a construct validation study using attachment theory. *J. Pers. Assess.* 91 601–610. 10.1080/00223890903230931 19838910

[B8] Cecchini M. Aceto P. Altavilla D. Palombo L. Lai C. (2013). The role of the eyes in processing an intact face and its scrambled image: a dense array ERP and low-resolution electromagnetic tomography (sLORETA) study. *Soc. Neurosci.* 8 314–325. 10.1080/17470919.2013.797020 23706064

[B9] Cecchini M. Iannoni M. E. Pandolfo A. L. Aceto P. Lai C. (2015). Attachment style dimensions are associated with brain activity in response to gaze interaction. *Soc. Neurosci.* 10 282–293. 10.1080/17470919.2014.998344 25568957

[B10] Clark L. A. Watson D. (1995). Constructing validity: basic issues in objective scale development. *Psychol. Assess.* 7 309–319. 10.1037/1040-3590.7.3.309 PMC6754793 30896212

[B11] Dan O. Raz S. (2012). Adult attachment and emotional processing biases: an event-related potentials (ERPs) study. *Biol. Psychol.* 91 212–220. 10.1016/j.biopsycho.2012.06.003 22732315

[B52] Electrical Geodesics Inc. (2007). *GeoSource Technical Manual*. Eugene: EGI.

[B12] Exner J. E. Jr. (1989). Searching for projection in the Rorschach. *J. Pers. Assess.* 53 520–536. 10.1207/s15327752jpa5303_9 2778616

[B13] Exner J. E. (2003). *The Rorschach: A Comprehensive System*, 4th Edn. New York, NY: John Wiley.

[B14] Feeney J. Noller P. Hanrahan M. (1994). *Assessing Adult Attachment.* New York, NY: Guilford Press.

[B15] Foley P. Kirschbaum C. (2010). Human hypothalamus-pituitary-adrenal axis responses to acute psychosocial stress in laboratory settings. *Neurosci. Biobehav. Rev.* 35 91–96. 10.1016/j.neubiorev.2010.01.010 20109491

[B16] Fonagy P. Luyten P. (2009). A developmental, mentalization-based approach to the understanding and treatment of borderline personality disorder. *Dev. Psychopathol.* 21 1355–1381. 10.1017/S0954579409990198 19825272

[B17] Fossati A. Feeney J. A. Donati D. Donini M. Novella L. Bagnato M. (2003). On the dimensionality of the Attachment Style Questionnaire in Italian clinical and nonclinical subjects. *J. Soc. Pers. Relatsh.* 20 55–79. 10.1177/02654075030201003

[B18] Fossati A. Feeney J. A. Grazioli F. Borroni S. Acquarini E. Maffei C. (2007). *L’Attachment Style Questionnaire (ASQ).* Milano, MI: Raffaello Cortina Editore.

[B19] Gilboa A. (2004). Autobiographical and episodic memory-one and the same? Evidence from prefrontal activation in neuroimaging studies. *Neuropsychologia* 42 1336–1349. 10.1016/j.neuropsychologia.2004.02.014 15193941

[B20] Giromini L. Porcelli P. Viglione D. J. Parolin L. Pineda J. A. (2010). The feeling of movement: EEG evidence for mirroring activity during the observations of static, ambiguous stimuli in the Rorschach cards. *Biol. Psychol.* 85 233–241. 10.1016/j.biopsycho.2010.07.008 20654683

[B21] Hünefeldt T. Laghi F. Ortu F. Belardinelli M. O. (2013). The relationship between ‘theory of mind’ and attachment-related anxiety and avoidance in Italian adolescents. *J. Adolesc.* 36 613–621. 10.1016/j.adolescence.2013.03.012 23595130

[B22] Kim S. H. Hamann S. (2007). Neural correlates of positive and negative emotion regulation. *J. Cogn. Neurosci.* 19 776–798. 10.1162/jocn.2007.19.5.776 17488204

[B23] Koban L. Pourtois G. Vocat R. Vuilleumier P. (2010). When your errors make me lose or win: event-related potentials to observed errors of cooperators and competitors. *Soc. Neurosci.* 5 360–374. 10.1080/17470911003651547 20349391

[B24] Lai C. Pellicano G. R. Ciacchella C. Guidobaldi L. Altavilla D. Cecchini M. (2020). Neurophysiological correlates of emotional face perception consciousness. *Neuropsychologia* 146:107554. 10.1016/j.neuropsychologia.2020.107554 32652090

[B25] Lamm C. Decety J. Singer T. (2011). Meta-analytic evidence for common and distinct neural net- works associated with directly experienced pain and empathy for pain. *Neuroimage* 54 2492–2502. 10.1016/j.neuroimage.2010.10.014 20946964

[B26] Lerner P. M. (1990). Rorschach assessment of primitive defenses: A Review. *J. Pers. Assess.* 54 30–46. 10.1080/00223891.1990.9673971 2179520

[B27] Lieberman M. D. (2007). Social cognitive neuroscience: a review of core processes. *Annu. Rev. Psychol.* 58 259–289. 10.1146/annurev.psych.58.110405.085654 17002553

[B28] Luciani M. Cecchini M. Altavilla D. Palombo L. Aceto P. Ruggeri G. (2014). Neural correlate of the projection of mental states on the not-structured visual stimuli. *Neurosci. Lett.* 573 24–29. 10.1016/j.neulet.2014.05.008 24831184

[B29] McGrath R. E. (2008). The Rorschach in the context of performance-based personality assessment. *J. Pers. Assess.* 90 465–475. 10.1080/00223890802248760 18704805

[B30] McRae K. Hughes B. Chopra S. Gabrieli J. D. E. Gross J. J. Ochsner K. N. (2010). The neural bases of distraction and reappraisal. *J. Cogn. Neurosci.* 22 248–262. 10.1162/jocn.2009.21243 19400679 PMC4136451

[B31] Meng X. L. Rosenthal R. Rubin D. B. (1992). Comparing correlated correlation coefficients. *Psychol. Bull.* 111 172–175. 10.1037/0033-2909.111.1.172

[B32] Meyer G. J. Kurz J. E. (2006). Advancing personality assessment terminology: Time to ritire “objective” and “projective” as personality test description. *J. Pers. Assess.* 87 223–225. 10.1207/s15327752jpa8703_01 17134328

[B33] Meyer G. J. Viglione D. J. Mihura J. L. Erard R. E. Erdberg P. (2011). *Rorschach Performance Assessment System: Administration, Coding, Interpretation, and Technical Manual.* Toledo, OH: Rorschach Performance Assessment System.

[B34] Mihura J. L. Meyer G. J. Dumitrascu N. Bombel G. (2013). The validity of individual Rorschach variables: systematic reviews and meta-analyses of the Comprehensive System. *Psychol. Bull.* 139 548–605. 10.1037/a0029406 22925137

[B35] Mikulincer M. Shaver P. R. (2007). *Attachment in Adulthood: Structure, Dynamics, and Change.* New York, NY: The Guilford Press.

[B36] Pianowski G. Meyer G. J. Villemor-Amaral A. E. (2016). Potential Projective Material on the Rorschach: Comparing Comprehensive System Protocols to Their Modeled R-Optimized Administration Counterparts. *J. Pers. Assess.* 98 398–407. 10.1080/00223891.2016.1147451 26963932

[B37] Pineda J. A. Giromini L. Porcelli P. Parolin L. Viglione D. J. (2011). Mu suppression and human movement responses to the Rorschach test. *Neuroreport* 22 223–226. 10.1097/WNR.0b013e328344f45c 21346645

[B38] Porcelli P. Giromini L. Parolin L. Pineda J. A. Viglione D. J. (2013). Mirroring activity in the brain and movement determinant in the Rorschach test. *J. Pers. Assess.* 95 444–456. 10.1080/00223891.2013.775136 23495976

[B39] Rolls E. T. (2015). Limbic systems for emotion and for memory, but no single limbic system. *Cortex* 62 119–157. 10.1016/j.cortex.2013.12.005 24439664

[B40] Rorschach H. (1921). *Psychodiagnostik.* Bern, BE: Bircher.

[B41] Serafini G. Pompili M. Borgwardt S. Houenou J. Geoffroy P. A. Jardri R. (2014). Brain changes in early-onset bipolar and unipolar depressive disorders: a systematic review in children and adolescents. *Eur. Child Adolesc. Psychiatry.* 23 1023–1041. 10.1007/s00787-014-0614-z 25212880

[B42] Shaver P. R. Mikulincer M. (2008). *An Overview of Adult Attachment Theory.* New York, NY: Guilford Press.

[B43] Solano P. Ustulin M. Pizzorno E. Vichi M. Pompili M. Serafini G. (2016). A Google-based approach for monitoring suicide risk. *Psychiatry Res.* 246 581–586. 10.1016/j.psychres.2016.10.030 27837725

[B44] Suzuki W. A. (1996). The anatomy, physiology and functions of the perirhinal cortex. *Curr. Opin. Neurobiol.* 6, 179–186. 10.1016/S0959-4388(96)80071-7 8725959

[B45] Vrtička P. Bondolfi G. Sander D. Vuilleumier P. (2012). The neural substrates of social emotion perception and regulation are modulated by adult attachment style. *Soc. Neurosci.* 7 473–493. 10.1080/17470919.2011.647410 22217336

[B46] Vrtička P. Sander D. Vuilleumier P. (2011). Effects of emotion regulation strategy on brain responses to the valence and social content of visual scenes. *Neuropsychologia* 49 1067–1082. 10.1016/j.neuropsychologia.2011.02.020 21345342

[B47] Vrtička P. Vuilleumier P. (2012). Neuroscience of human social interactions and adult attachment style. *Front. Hum. Neurosci.* 6:212. 10.3389/fnhum.2012.00212 22822396 PMC3398354

[B48] Weiß C. H. (2007). *Statistica, version 8.* Tulsa, OK: Statsoft, inc.

[B49] Weiner I. B. (2003). *Principles of Rorschach Interpretation*, 2nd Edn. Mahwah, NJ: Lawrence Erlbaum Associates.

[B50] Weiner I. B. (2018). Society for Personality Assessment/Journal of Personality Assessment: A History. *J. Pers. Assess.* 100 2–15. 10.1080/00223891.2017.1394869 29261358

[B51] Yoto A. Katsuura T. Iwanaga K. Shimomura Y. (2007). Effects of object color stimuli on human brain activities in perception and attention referred to EEG alpha band response. *J. Physiol. Anthropol.* 26 373–379. 10.2114/jpa2.26.373 17641457

